# Supplementary figures and images for: Wolbachia action in the sperm produces developmentally deferred chromosome segregation defects during the Drosophila mid-blastula transition
Source: eLife. 2022 Sep 23;11:e81292. doi: 10.7554/eLife.81292 (PMC9507124; doi:10.7554/eLife.81292)

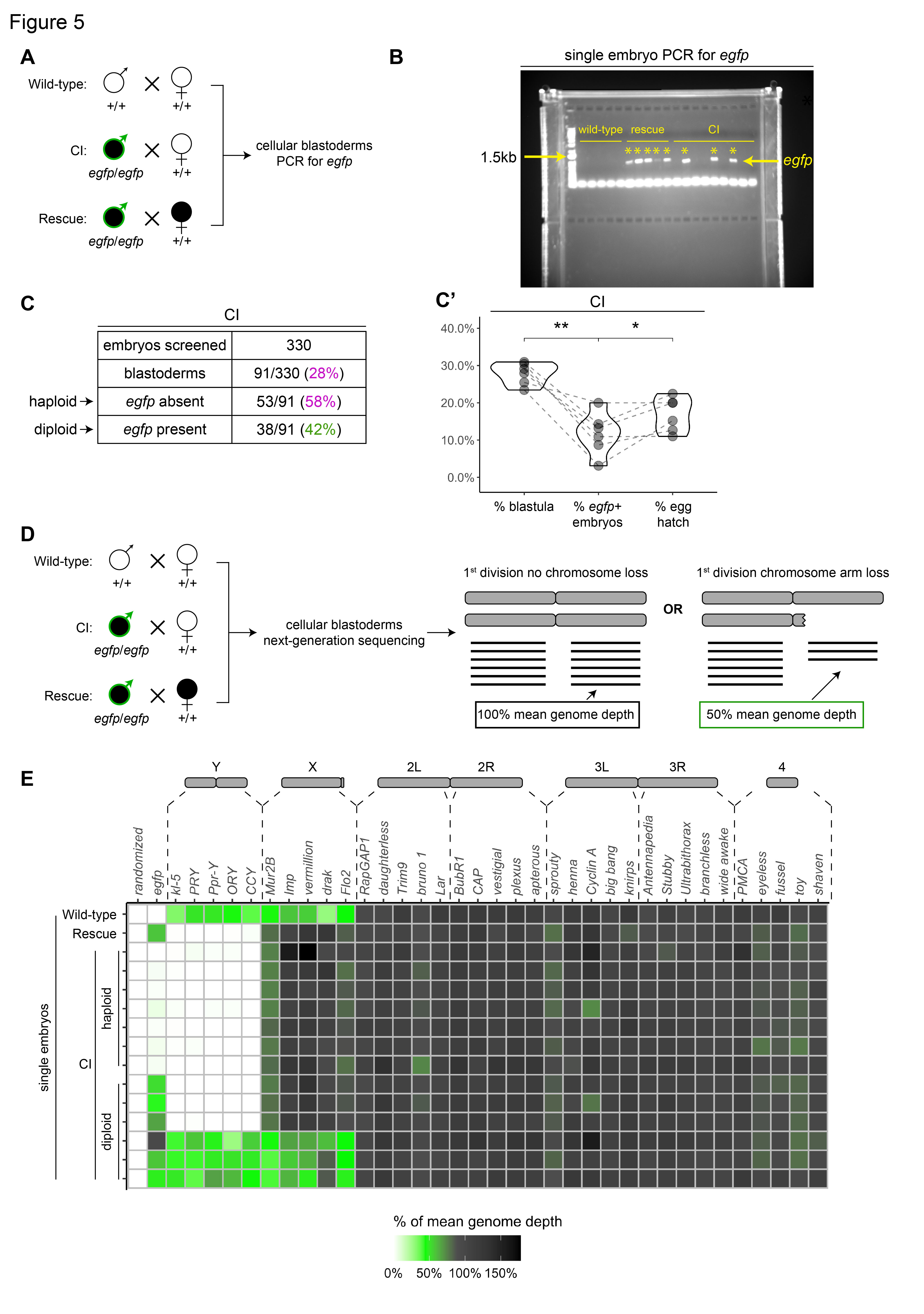

Supplement: Figure 5—source data 1. — Raw gel image used to create Figure 5B (raw unedited gel) and Figure 5 built with the raw unedited gel (Figure 5 with uncropped gel). [file elife-81292-fig5-data1.zip › Figure 5-source data 1/Figure 5-source data 1-Figure 5 with uncropped gel.jpg]

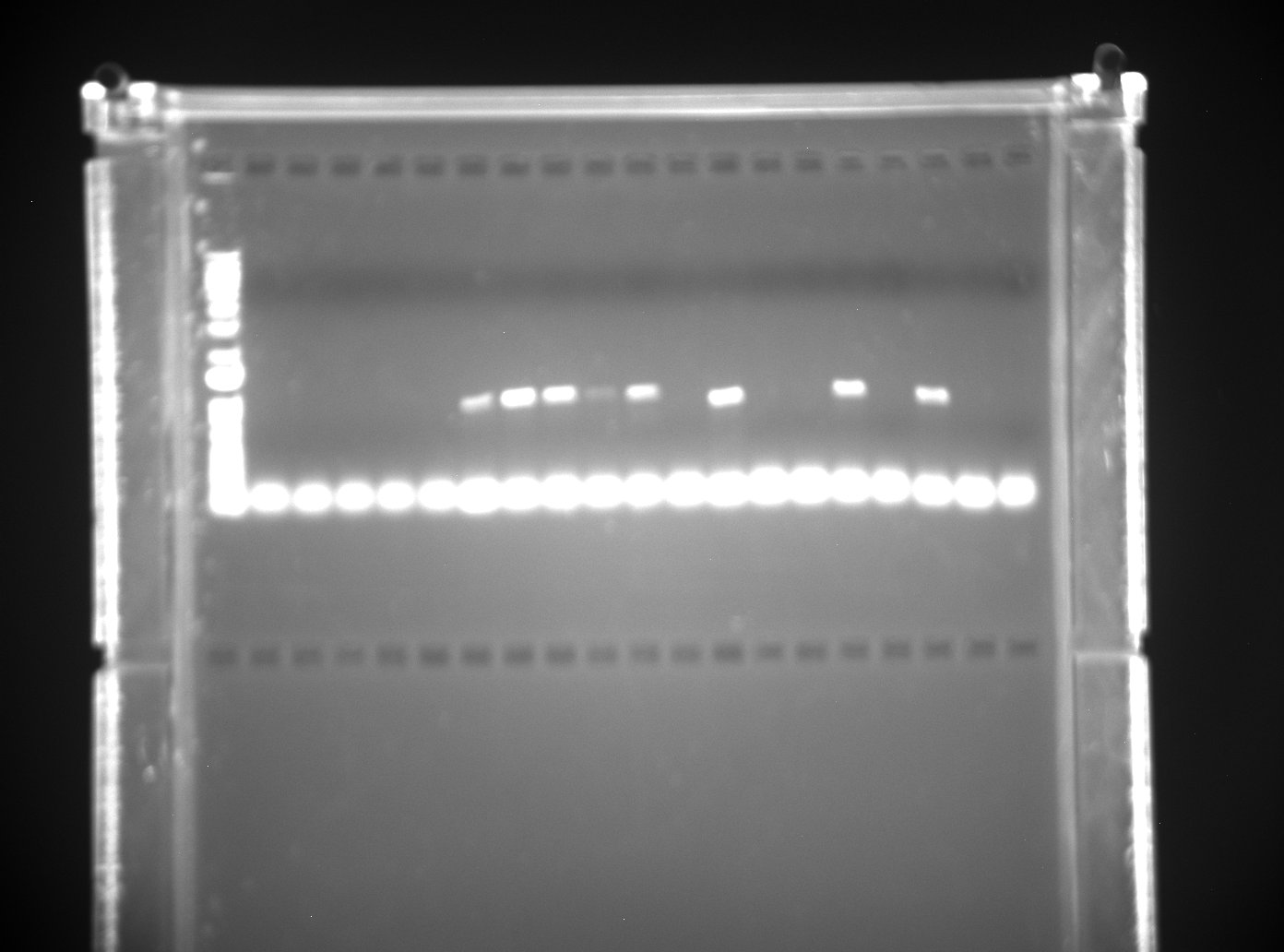

Supplement: Figure 5—source data 1. — Raw gel image used to create Figure 5B (raw unedited gel) and Figure 5 built with the raw unedited gel (Figure 5 with uncropped gel). [file elife-81292-fig5-data1.zip › Figure 5-source data 1/Figure 5-source data 1-raw unedited gel.jpg]
